# The Wessex Fit-4-Cancer Surgery Trial (WesFit): a protocol for a factorial-design, pragmatic randomised-controlled trial investigating the effects of a multi-modal prehabilitation programme in patients undergoing elective major intra–cavity cancer surgery

**DOI:** 10.12688/f1000research.55324.1

**Published:** 2021-09-21

**Authors:** Malcolm West, Andrew Bates, Chloe Grimmett, Cait Allen, Richard Green, Lesley Hawkins, Helen Moyses, Samantha Leggett, Denny Z H Levett, Sally Rickard, Judit Varkonyi-Sepp, Fran Williams, Stephen Wootton, Matthew Hayes, Micheal P W Grocott, Sandy Jack

**Affiliations:** 1 NIHR Southampton Biomedical Research Centre, University Hospital Southampton NHS Foundation Trust, Southampton, SO16 6YD, UK; 2 School of Cancer Sciences, Faculty of Medicine, University of Southampton, Southampton, SO16 6YD, UK; 3 School of Health Sciences, University of Southampton, Southampton, SO22 1BJ, UK; 4 Wessex Cancer Trust, Registered charity 1110216, Chandlers Ford, SO53 2GG, UK; 5 Anaesthetic Department (Royal Bournemouth Site), University Hospitals Dorset, Bournmouth, BH77DW, UK; 6 Critical Care/Anaesthesia and Perioperative Medicine Research Unit, University Hospital Southampton NHS Foundation Trust, Southampton, SO16 6YD, UK; 7 School of Clinical and Experimental Science, Faculty of Medicine, University of Southampton, Southampton, SO16 6YD, UK; 8 Wessex Cancer Alliance, Oakley Road, Southampton, SO16 4GX, UK; 9 School of Human Development and Health, Faculty of Medicine, University of Southampton, Southampton, SO16 6YD, UK

**Keywords:** Surgery, Prehabilitation, Cardiopulmonary Exercise Test, Exercise, Physical activity, Psychological, Wellbeing, Neoadjuvant, Chemotherapy, Chemoradiotherapy, Outcome

## Abstract

**Background: **Surgical resection remains the primary curative treatment for intra-cavity cancer. Low physical fitness and psychological factors such as depression are predictive of post–operative morbidity, mortality and length of hospital stay. Prolonged post-operative morbidity is associated with persistently elevated risk of premature death. We aim to investigate whether a structured, responsive exercise training programme, a psychological support programme or combined exercise and psychological support, delivered between treatment decision and major intra-cavity surgery for cancer, can reduce length of hospital stay, compared with standard care.

**Methods: **WesFit is a pragmatic **, **2x2 factorial-design, multi-centre, randomised-controlled trial, with planned recruitment of N=1560. Participants will be randomised to one of four groups. Group 1 (control) will receive usual pre-operative care, Group 2 (exercise) patients will undergo 2/3 aerobic, high-intensity interval training sessions per week supervised by personal trainers. Group 3 (psychological support) patients are offered 1 session per week at a local cancer support centre. Group 4 will receive both exercise and psychological support. All patients undergo baseline and pre-operative cardiopulmonary exercise testing, complete self-report questionnaires and will be followed up at 30 days, 12 weeks and 12 months post-operatively. Primary outcome is post-operative length-of-stay. Secondary outcomes include disability-adjusted survival at 1-year postoperatively, post-operative morbidity, and health-related quality of life. Exploratory investigations include objectively measured changes in physical fitness assessed by cardiopulmonary exercise test, disease-free and overall mortality at 1-year postoperatively, longer-term physical activity behaviour change, pre-operative radiological tumour regression, pathological tumour regression, pre and post-operative body composition analysis, health economics analysis and nutritional characterisation and its relationship to post-operative outcome.

**Conclusions: **The WesFit trial will be the first randomised controlled study investigating whether an exercise training programme +/- psychological intervention results in improvements in clinical and patient reported outcomes in patients undergoing major inter-cavity resection of cancer.

**ClinicalTrials.gov registration: **NCT03509428 (26/04/2018)

## Introduction

The number of new cancer cases per year is expected to rise to 23.6 million by 2030. Depending on the cancer cohort, major curative cancer surgery is associated with post-operative morbidity in up to 50% of gastrointestinal cancer patients and up to 60% in pancreatic cancer patients especially after neoadjuvant cancer treatments. ^1– 3^ Improved surgical, oncological and anaesthetic techniques, enhanced recovery pathways and perioperative care have delivered consistent improvements in length of hospital stay, in-hospital morbidity and readmission rates after major surgery. However, over half of patients over the age of 60 years after major abdominal surgery live with reduced functional capacity, physical fitness and quality of life (QoL), with a significant proportion never regaining pre-operative fitness or independent living. ^4, 5^ Our group and others have described the association between reduced pre-operative physical fitness ^6– 11^ (and its decline with neoadjuvant cancer treatments) and poor post-operative outcomes in upper and lower gastrointestinal cancer patients. Furthermore, we have reported on the associations between reduced physical fitness and reduced mitochondrial function, ^12^ QoL ^13, 14^ and physical activity ^15^ after cancer treatments before surgery.

Cancer prehabilitation is a novel process that occurs between the time of cancer diagnosis and continues throughout the cancer treatment pathway. The time before cancer diagnosis and surgery is an emotionally salient time where patients are receptive to changes in behaviour regarding their nutrition, fitness and psychological coping. Physical fitness, nutritional and psychological multimodal prehabilitation are targeted, tailored interventions with the aim to prevent, minimise and/or rescue the severity of anticipated treatment-related impairments that may cause significant disability when recovering from major cancer surgery. ^16^ Given the multi-system impact of cancer and its treatment(s), prehabilitation interventions have adopted a ‘multimodal approach’, that may be defined as the incorporation of two or more intervention components specifically selected for their potential cumulative or synergistic effects on health outcomes. Reviews of prehabilitation in surgical oncology identify many limitations in the current evidence, yet acknowledge encouraging findings including improved fitness, endurance time, length of hospital stay, surgical complication rates, and health-related QoL with prehabilitation interventions. ^17– 26^ Recently, multinational consensus statements jointly from Macmillan Cancer Support, the Royal College of Anaesthetists and the National Institute for Health Research in the United Kingdom, ^27^ Exercise and Sports Science Australia ^28, 29^ and the Academy of Medical Royal Colleges in the United Kingdom ^30^ aim to advance the care provision, inform a change in policy, inform service provision, and implement a practice to benefit people living with cancer. Specifically, effective prehabilitation using multimodal physical activity, exercise, nutrition and psychological support, underpinned by behaviour change support, to improve cancer outcomes is advocated. Thus far the evidence is mostly based on uni-modal interventions, for example exercise interventions to improve physical fitness and post-operative complications ^20, 22, 31– 34^ and respiratory interventions to improve pulmonary specific morbidity, ^26^ however literature utilising multimodal interventions (mostly exercise and nutrition) to improve outcomes is now emerging. ^18, 19, 35, 36^

To date exercise prehabilitation trials have focused on in-hospital training with little consideration for sustainable models of delivery. There has also been a lack of inclusion of behavioural science to facilitate engagement and longer-term behaviour change post-surgery. Furthermore, increasingly, evidence suggests that psychological factors impact physiological and psychological outcomes in both the short and long term, with implications for recovery from surgery, QoL and re-attainment of independent living. ^37^ A systematic review of psychological prehabilitation before cancer surgery suggests such interventions can positively impact QoL and psychological outcomes (such as distress and anxiety). However prehabilitation studies including a psychological component have tended to be small, therefore, an evaluation of a psychological intervention together with an exercise intervention as part of a multimodal prehabilitation programme in cancer patients is urgently needed. ^23^

## Protocol

### Study design and setting

**Figure 1.**
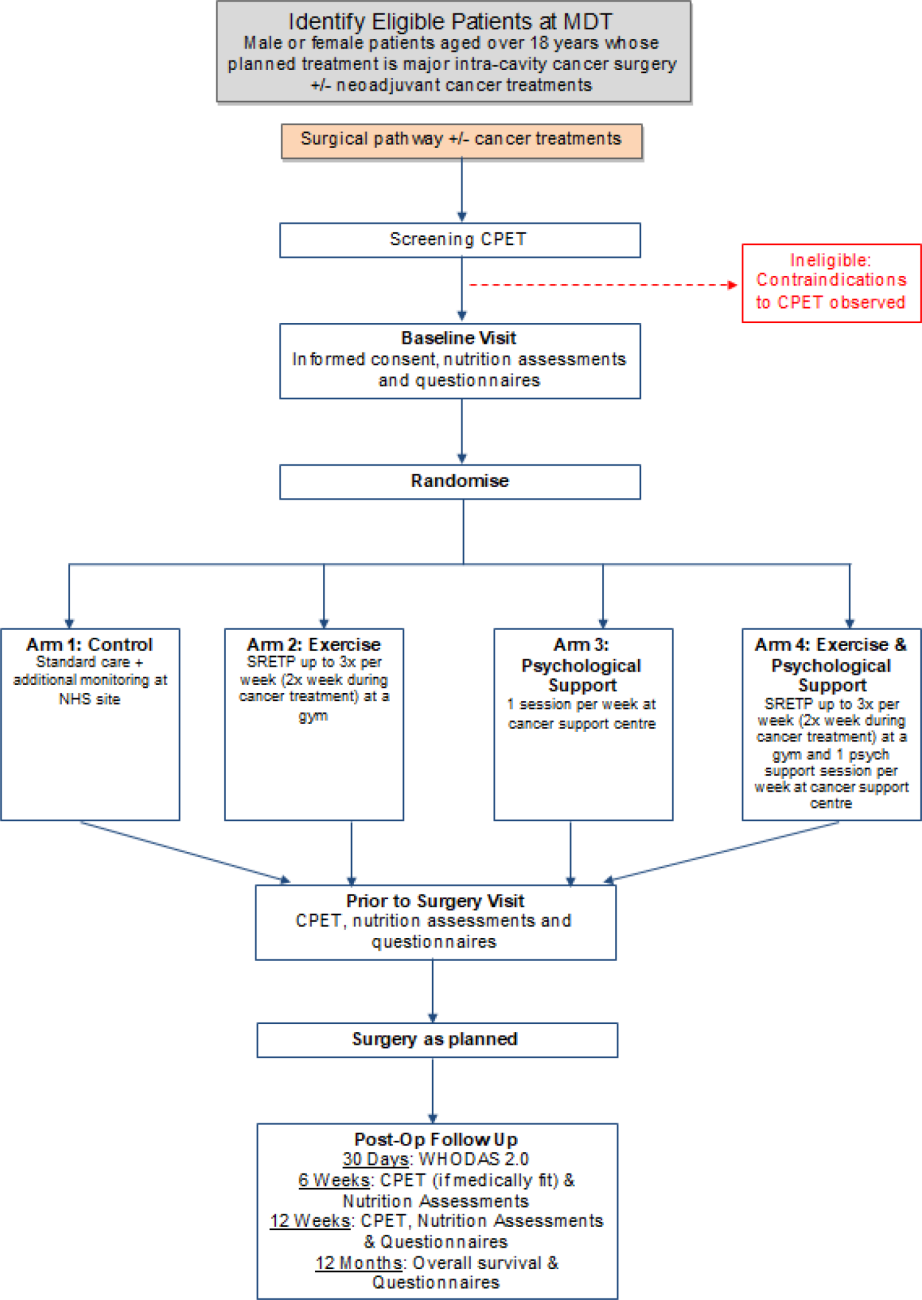
Trial summary diagram. MDT: multidisciplinary team, CPET: cardiopulmonary exercise test, SREPT: structured responsive exercise training programme, WHODAS: World Health Organisation Disability Adjusted Survival.

The Wessex Fit-4 Cancer Surgery trial (WesFit) is a multi-centre, 2 × 2 factorial design, randomised-controlled, single blind, phase III clinical trial designed to compare the effect of structured prehabilitation programmes, against standard pre-operative care on patient outcome following major intra-cavity surgery for cancer ( Figure 1). Participant recruitment, assessment and intervention are organised by NHS Hospital trusts who oversee referral to our community partners in local gymnasiums (exercise and behaviour change intervention) and Cancer Support centres (psychological intervention). The trial was registered with clinicaltrials.gov (NCT03509428) on April 26th, 2018. This protocol follows the SPIRIT guidelines – see *Reporting guidelines*. ^56^

### Study objectives

Primary objective: investigate whether a multimodal exercise, and psychological support prehabilitation programme, performed in a community-based setting prior to major cancer surgery, (± neoadjuvant cancer treatments) will result in a clinically significant difference (1-day reduction) in post-operative in-hospital length of stay) when compared to a control group.

Secondary objectives: investigate whether the WesFit programme performed prior to major cancer surgery ± neoadjuvant cancer treatments:

i. Improves disability free survival as measured by World Health Organisation (WHO) Disability Adjusted Survival v2.0
ii. Improves post-operative morbidity ^38^ as measured by post-operative morbidity score (POMS) and Clavien-Dindo-Demartines (CD) ^39^/comprehensive complication index (CCI) scores ^40^
iii. Improves health related QoL as measured by EQ-5D-5L ^41^ and EORTC-QLQ-C30. ^42^

Exploratory objectives include:

i. Improve overall survival and disease-free at one-year post-surgery
ii. Improve selected cardiopulmonary exercise test (CEPT) physiological variables
iii. Improve long-term physical activity behaviour
iv. Demonstrate cost effectiveness determined by health economics analysis
v. Improve radiological markers of body composition
vi. Improve radiological tumour regression grade (TRG)
vii. Improve pathological tumour regression grade (yTRG)
viii. Improve psychological outcomes including confidence to self-manage illness, anxiety and depression.
ix. Improve body composition measured by computer tomography and bioelectrical impedance analyses
x. Improve nutritional and micronutrient status measured by micronutrient blood analysis

### Eligibility criteria

Patients will be eligible for WesFit if they are over 18 years old and are scheduled to have major, intra-cavity cancer surgery with a curative intent. These are defined as thoracic, colorectal (including anal and neuroendocrine tumours), oesophagogastric (including neuroendocrine tumours), urological (including prostate, bladder and renal tumours), head and neck (including nasopharyngeal, laryngeal, pharyngeal and oral) and hepatobiliary (including pancreatic, gall bladder and neuroendocrine tumours). Treatment includes surgery alone or surgery combined with cancer treatments (including but not limited to neoadjuvant chemotherapy, chemoradiotherapy or immunotherapies). All patients deemed by the multidisciplinary team (MDT) as potentially curable or undergoing neoadjuvant cancer treatments with curative intent prior to restaging and surgery will be included.

Exclusion criteria includes patients with a tumour that is considered surgically non-resectable, having absolute or relative contraindications to completing a CPET, ^43^ patients unable to perform CPET due to other coexisting acute illness or conditions (e.g. lower limb dysfunction), patients declining surgery or planned neoadjuvant treatment, if their weight exceeds 160 kg and patients unable to give informed consent.

### Recruitment and randomisation

Consecutive, potentially eligible patients will be identified at MDT meetings and approached with patient information sheets at surgical/oncological outpatient clinic appointments. If the patient chooses to participate in this trial, they will undergo a screening CPET. This constitutes part of standard clinical care in some NHS hospitals. Once reviewed by a senior clinician, final eligibility is confirmed, and written informed consent can be obtained for trial participation.

Patients will be randomised 1:1:1:1 to one of four groups. The study design is a 2 x 2 factorial design. The randomisation will be performed using ALEA ^TM^ from FormsVision, an online software not under the control of the study team. Patients will be randomised by ALEA ^TM^ by minimisation to the multimodal interventions or the control arm and stratified according to tumour type, hospital site, gender, neoadjuvant cancer therapy and age. Group 1 is control (routine care), group 2 is exercise alone, group 3 is psychological support alone and group 4 is a multimodal exercise, and psychological support.

WesFit began recruitment in April 2018 and at the time of publishing was paused due to coronavirus disease 2019 (COVID-19) lockdown restrictions.

### Interventions

**Exercise intervention:** The exercise-training programme is consistent with the FITT principle (frequency, intensity, time and type), as advised by a panel of international experts and patient representatives. The exercise intervention has been shown by our group to be safe, feasible and tolerable in locally advanced rectal cancer patients following neoadjuvant chemoradiotherapy. ^31, 39^ Patients will participate in a prescribed, supervised, aerobic high-intensity interval, structured, responsive, exercise training programme (SRETP) on an electronically-braked cycle ergometer (Ergoselect Cloud bike). Participants will undertake 3-sessions per week (2-sessions per week if undergoing neoadjuvant treatments), from recruitment to surgical resection. These will occur within community gymnasia, unless precluded by safety concerns due to clinical condition. High-risk patients can exercise within a hospital setting. The high-intensity interval training (HIIT) comprises of an initial 5 minutes of unloaded pedalling. This is followed by 3 minutes at moderate intensity and 2 minutes at severe intensity. Moderate intensity exercise refers to the patient’s power output at 80% of oxygen uptake (VO _2_) at the anaerobic threshold, (80%AT) derived at baseline CPET. Severe exercise intensity occurs at the patient’s power output, at 50% of the difference between the VO _2_ at AT and VO _2_ Peak (50%∆) also accounting for 2/3 of the ramp work rate. These 5-minute intervals will be repeated 6 times, followed by 5 minutes of unloaded pedalling. The entire session lasts for 40 minutes. If the patient is receiving neoadjuvant treatment the entire session lasts for 30 minutes, with the 5-minute intervals repeated 4 times rather than 6 times. The full exercise programme is reported according to the Template for Intervention Description and Replication (TIDiER) checklist for exercise. The checklist is available in *Extended data*. ^56^

As part of the exercise intervention personal trainers delivering the supervised exercise intervention will receive training in behaviour change support in the form of Healthy Conversation Skills (HCS). HCS is a brief intervention, developed to equip health and social care practitioners with the skills to support improvements in diet and physical activity in their patients/clients. ^44^ Informed by principles of motivational interviewing and social cognitive theory it is an empowering, client-centred, solution-focused approach to support behaviour change. Self-efficacy, a central construct of Bandura’s social cognitive theory, ^45^ describes a person’s belief in their abilities to perform a given task. Self-efficacy is also considered to be a prerequisite to an individual experiencing a sense of control. Evidence suggests that self-efficacy is a mediator of exercise behaviour in clinical populations and a predictor of exercise adherence. ^46^ See Table 1 for included behaviour change techniques as per the behaviour change technique taxonomy. ^47^ Personal trainers work with participants throughout the intervention to increase motivation, self-efficacy and support planning for continued unsupervised exercise after surgery. Personal trainers have telephone consultations at three and six months after surgery to support long-term engagement in independent physical activity. The behaviour change intervention is reported according to the TIDiER checklist in *Extended data*. ^56^

**Table 1.** Behaviour change techniques (BCT) coded to the BCT taxonomy (BCTT V1).

BCT label	BCT no. (BCTT v1)	Example Intervention component
Goal setting (behaviour)	1.1	Participants set goals for independent exercise engagement following surgery.
Problem solving	1.2	Trainers use the SMARTER goal setting sheets to prompt the participant to analyse factors that might get in the way of them achieving a goal and how it can be overcome.
Action planning	1.4	Trainers use SMARTER goal setting sheets to prompt detailed specification of goals including day of the week and time that they will perform a particular behaviour to be performed in the early recovery period after surgery.
Review behaviour goal(s)	1.5	Trainers will review behaviour goal(s) with the participants and modifies them collaboratively as necessary, e.g. setting an easier goal if the previous goal was not achievable.
Feedback on behaviour	2.2	The trainer and participant will reflect and discuss changes to exercise behaviour, particularly when exercising independently after surgery.
Self-monitoring of behaviour	2.3	Participants will be encouraged to keep a diary of their independent exercise.
Social support (unspecified)	3.1	The trainer will provide praise and encouragement throughout the trial.
Instruction on how to perform a behaviour	4.1	The trainer will provide specific instructions during the structured exercise sessions.
Graded tasks	8.7	The trainer will work with participants to start with easy to achieve independent exercise goals, gradually increasing the difficulty overtime.
Credible source	9.1	The trainer presents as a credible source with in-depth understanding of the benefits of exercise following a cancer diagnosis.
Verbal persuasion about capability	15.1	The trainer will reassure participants that the exercise programme is based on their fitness levels and is achievable.
Focus on past success	15.3	The trainer will encourage the participant to review progress made over the course of the trial.

**Psychological support:** Psychological support will be provided in the form of counselling. Counselling will be delivered by counsellors experienced in working with people affected by cancer. Counsellors are members of the British Association of Counselling and Psychotherapy with a minimum qualification of a Diploma in Counselling and Psychotherapy. The processes reflect the best practice currently delivered by cancer support centre staff. Participants will be offered weekly one-to-one consultations lasting up to 1 hour, allowing them to explore any issues/concerns they are experiencing, including (but not limited to) ways of coping with their reaction to cancer, family and relationship issues, anxiety and distress. After each session, counsellors will complete a checklist indicating counselling techniques used. The psychological support intervention is reported according to the TIDiER checklist in *Extended data*. ^56^

Adherence will be monitored throughout the trial. The trial team will receive automatic uploads of exercise adherence from the card- and cloud-based systems and attendance (or not) at counselling sessions will be logged.

**Control group:** The control group will receive routine pre- and post-operative care with additional assessments common to all groups, but no intervention outside of routine care.

## Outcome measures

Baseline assessment will occur as close as possible to the MDT treatment decision. All patients repeat assessments immediately prior to surgery. Patients undergoing neoadjuvant cancer treatment (e.g. chemoradiotherapy for locally advanced rectal cancer patients) will undergo repeat CPET every 4-weeks (depending on site availability), in order to assess continued eligibility and to moderate training intensities according to physiological adaptation. Post-operative assessment occurs during hospital admission, at 30 days post-surgery, at 6- and 12- weeks post-surgery and 12-months post-surgery. The schedule of observations and procedures can be found in the *Extended data*. ^56^

## Primary outcome

Length of stay is defined as the number of days a patient stays in hospital following surgery. It is calculated by subtracting the date of surgery from the date of discharge. The date of surgery is defined as day 0 of a patient’s post-operative hospital stay.

## Secondary outcomes

i. World Health Organisation Disability Adjusted Survival 2.0 (WHODAS 2.0) - The 36 item WHODAS 2.0 questionnaire will be completed at baseline, prior to surgery, day-30, week-12 and 12-months following date of surgery.
ii. Post-operative morbidity will be determined by the post-operative morbidity survey (POMS), ^48^ the highest in-hospital morbidity achieved according to the Clavien-Dindo-Demartines (CD) score ^39^ and the comprehensive complication index (CCI) score. ^40^ Patients’ POMS will be characterised on day 3, 5, 7 and 15, while patient remains hospitalised. The POMS 18-item survey will be used to address nine domains of postoperative morbidity (pulmonary, infectious, renal, gastrointestinal, cardiovascular, neurological, wound complication, haematological and pain). On day of discharge, patient’s surgical complications (if any) will be graded using the CD classification of surgical complications This classification is used to assess overall hospital morbidity following surgical procedures. Patients are graded as 0 (no complications) or Grade I-V based on the level of complication, including the number of organ system involvement. Grade V is defined as death of a patient. A record of the CCI – an update of the CD classification will also be collected.
iii. Health related QoL will be assessed using the EQ-5D-5L ^40^ and the EORTC-QLQ-C30. ^41^ EQ-5D-5L is a standardised measure of health status which provides a simple, generic measure of health for clinical and economic appraisal. There are 5 domains (mobility, self-care, usual activities, pain/discomfort, anxiety/depression) each with 5 levels of health. It also includes a visual analogue scale which asks the respondent to rate their health from 0 (worst imaginable) to 100 (best imaginable health). Cancer specific, health-related QoL will be measured using the EORTC-QLQ-C30. This EORTC-QLQ-C30 measures physical, role, emotional, social and cognitive functioning, as well as global QoL and three symptoms; fatigue, pain and nausea/vomiting. For all scales scores range from 0-100. For global and functional scales higher scores reflect favorable QoL, whereas higher symptom scales score indicate more symptoms.

## Exploratory outcomes

i. Overall and disease-free survival at 1-year post-surgery
ii. VO _2_ at AT (anaerobic threshold), VO _2_ at peak, and VE-VCO _2_ slope. Other CPET variables will be reported and analysed as per previous publications. ^8, 12, 32, 38^
iii. Radiological makers of body composition will be measured from routine abdominal computed tomography (CT) scans at L3. ^48, 49^
iv. Radiological tumour downsizing/sizing/regression will be assessed using CT, magnetic resonance imaging (MRI) and positron emission tomography (PET) scans for all patients including those who have received neoadjuvant treatments. Clinical radiological tumor, node and metastasis (TNM) and tumour specific regression scores (RECIST ^50^) will be recorded after each clinical scan (baseline and re-staging).
v. Body composition will be measured using bioelectrical impedance analysis at baseline, immediately before surgery and at 6- and 12-weeks after surgery using the supplied SECA ^TM^ body composition analyser MBCA 515 weighing scale.
vi. Patients will undertake assessments through the trial in order to characterise nutritional status and relate to post-operative outcome, response to the trial interventions and response to neoadjuvant cancer treatments.
vii. Objective physical activity levels will be determined in a sub-sample of participants by ActiGraph ^TM^ GT9X link activity sensor, worn for 5 complete days, following baseline CPET, +/-during cancer therapies and at 12-weeks and 12-months following surgery.
viii. Quality-adjusted life years (QALY) will be used as a measure of health outcome for economic evaluation, incorporating both the quantity and the quality of patients’ lives. The EQ-5D-5L will be used to evaluate QALYs to evaluate both the morbidity gains and the mortality impact of prehabilitation in cancer patients. ^51^
ix. Anxiety and depression will be measured using the hospital anxiety and depression scale (HADS). ^52^
x. Self-efficacy (confidence) to self-manage chronic disease (SEMCD), will be measured using the Lorig SEMCD scale. ^53^
xi. Patient activation will be measured using the PAM (patient activation measure). ^54^

## Process evaluation

Qualitative in-depth semi-structured interviews will be conducted with patients enrolled in the trial and professionals involved in the delivery of the trial at two time points. Interviews of up to 1 hour will be conducted either face to face at a location convenient to the participant or by telephone depending on preference. A researcher with experience in qualitative interviewing and who is not part of intervention delivery will conduct the interviews. Firstly, patients from all arms of the study (N = 12) and key stakeholders; personal trainers, research nurses and counsellors (N = 5) will be interviewed regarding their experiences of the trial once the first 30 patients have reached 12 weeks post-surgery. The sample size is pragmatic based on the time and resource available early in the trial and will provide an opportunity for the research team to reflect on the experiences of patients and professionals involved in the trial, identifying barriers and facilitators to trial processes and implementation (professionals) and trial experiences (patients). If any amendments to the trial are deemed necessary, a substantial amendment will be submitted to the ethics committee and Health Research Authority (HRA) for approval.

Following completion of the trial structured interviews will be conducted with 25 patients (with representation from all 4 study arms) to understand patient experience of the trial and subsequent behaviour change. The sample size is will allow for purposive sampling with sampling characteristics including a range of age, sex, disease type, and whether or not they received neoadjuvant treatments. Interviews will also be conducted with stakeholders involved in the delivery of the trial, to include personal trainers, counsellors, research nurses (including other research team members) and members of clinical care teams, commissioners and cancer centre staff (N = 15). These interviews will seek to understand the barriers and facilitators to the implementation of the trial into clinical practice and within the community setting. All interviews will be audio-recorded, transcribed verbatim managed through NVIVO software (v12) and analysed using thematic analysis with an inductive approach. Normalisation process theory will inform data collection and analysis. ^55^

## Estimation of sample size

From previous studies, the median length of stay in the control group is estimated to be 7 days. To detect a clinically meaningful difference of a (significance level to detect a hazard ratio of 1.17 when the control group median in-patient time is 7 days) 1 day reduction in LOS with 85% power, alpha = 0.05, a sample size of 1560 participants will need to be recruited over 3 years, with a one year follow-up period. The sample size allows for a 20% drop-out.

Sample size calculations for 2×2 designs are based on the two main comparisons (i.e. exercise vs. no exercise and psychological support vs. no support). The trial sample size is the larger of these 2 comparisons, so in this case, the sample size calculation is powered on the psychological support comparison. The sample size calculation assumes that there is no interaction between the interventions.

## Statistical analysis plan

Descriptive statistics will be used to summarise the baseline demographic and clinical variables. For continuous variables, if the data are normally distributed, the mean and standard deviation will be calculated. If the data are not normally distributed, the median and interquartile range will be calculated. For categorical or binary variables, these will be summarised as frequency and percentage of total. There will be a variety of data consistency and quality checks performed at various stages of the data capture process, e.g. regular calibration and monitoring of measuring instruments, use of control standards in assays. All extreme values (mean +/- 3* standard deviation) and improbable values, as defined by clinical opinion will be investigated. In depth descriptions of these procedures exist in the trials data management plan, data management procedure and the data validation plan (code book) held by the sponsor. Outcomes assessors and statisticians will be blinded to trial arm until completion of analysis.

### Primary outcome

We will summarise, by group, any patients who were randomised, but did not have surgery, and reasons (i.e. death or withdrawal). We will also summarise by group the time between randomisation and surgery. Competing-risks survival regression will be used to model length of hospital stay. This allows for the fact that the participant may die in hospital, thus preventing the occurrence of the event of interest (discharge from hospital). As the time between randomisation and surgery will vary between participants, this will be included in the model. A multivariate model analysis will also be performed adjusting for clinically prognostic factors including age, gender, tumour type, T-stage and neoadjuvant treatment (yes/no). As a secondary analysis, we will perform the above including an interaction term in the model in order to test whether the effect of exercise differs according to whether psychological support is provided or not, although it is recognised that the power to detect any significant interactions in this number of patients will be low.

### Secondary outcomes

i. WHODAS total score, and each category score (cognition, mobility, self-care, interpersonal relationships, life activities and participation in society) will be summarised by intervention group at baseline, 30 days, 12 weeks and 12 months using mean (standard deviation) or median (interquartile range) depending on the normality of data.
ii. Disability free survival will be assessed by identifying whether the patient has a WHODAS score less than 25% at 1-year post surgery, and will be compared between treatment groups using logistic regression. A multivariate analysis may be performed adjusting for clinically prognostic factors, as specified for the primary endpoint.
iii. The Clavien-Dindo complication score is an ordinal variable with classification grades I, II, IIIa, IIIb, IVa, IVb, V. The wilcoxon-mann-whitney test will be used to compare between groups. If appropriate, ordinal logistic regression will be used to perform an analysis adjusted for important prognostic variables. The CCI score provides a measure of overall morbidity over the whole period following an intervention, which is reflected on a scale from 0 (no complication) to 100 (death). Data will be checked for Normality and summarised using mean (SD) or median (IQR) as appropriate. If the scores are normally distributed, a t-test will be used to compare groups, and linear regression modelling may be considered in order to adjust for baseline characteristics. For non-normal data, we will check whether logistic transformation improves normality. If not, the wilcoxon-mann-whitney test will be used. The proportion of patients with post-operative morbidity according to POMS will be summarised in each of the nine domains by intervention group/day. Due to the repeated nature of the data, mixed modelling will also be considered.
iv. The EQ 5D subscales and overall Health scale will be tabulated at each timepoint (baseline, pre-surgery, week-12 and 12-months post-surgery). Linear mixed modelling will be performed with overall health scale as the outcome variable, and intervention group and baseline health scale as explanatory variables to investigate the effect of intervention considering all timepoints. This will be repeated for the EORTC-QLQ-C30 global health score, and mixed effects ordered logistic regression will be considered for analysis of subscales.

## Patient and public involvement

Patient and public involvement has been included throughout. As part of the development of WesFit 2 focus groups and 1 interview were conducted with patients who had been encouraged to exercise prior to cancer treatment including: an individual who had undergone exercise in a community setting following general practitioner (GP) referral, a focus group (N = 12) of patients and carers who had taken part in clinical trials conducted by University Hospital Southampton (UHS) NHS Foundation Trust where they had performed in-hospital exercise training and a focus group (N = 2) of patients and cares who had attended the ‘Fit 4 surgery’ school at UHS. This informed the design of the trial such as the inclusion of support from personal trainers after surgery and the most appropriate language used to communicate the psychological element of the trial. These discussions also informed the managed process of community referral with links to the clinical teams clearly visible to patients to ensure they felt safe. Three patient representatives are included in the trial management group. They review and revise all patient facing documentation and trialled completion of all patient reported outcome measures. They were also consulted regarding the burden of the trial on participants. They will also help inform and facilitate future dissemination plans.

## Ethics and dissemination

The trial was initially authorised by London – Westminster Research Ethics Committee (REC reference 18/LO/0129) on 06/03/2018. Before a patient is randomised to the WesFit Trial, written informed consent will be obtained. When obtaining consent from a patient, the trial and the patient information sheet will be introduced in full. Written confirmation that the patient has given their consent to participate in the trial will be recorded by member of the research team according to local practice.

**The chief investigator and trial sponsor will have access to the full dataset.** Generalisable results will be published in scientific journals, incorporated into multi-disciplinary society guidelines and presented at cross-disciplinary international scientific conferences, patient groups, cancer charities and NIHR strategic partners.

## Monitoring and trial management

University Hospital Southampton NHS Foundation Trust is acting as sponsor for this trial. The sponsor will ensure that all regulatory policies adhered to in line with GCP and phamacovigilance policies. Day to day trial management, including site set-up, training and urgent consideration of safety concerns, will be the responsibility of the chief investigator. The trial management group will meet monthly and oversee the day to day running of the WesFit trial.

A project board will be responsible for all governance and finance frameworks and have oversight of the study conduct and management. The board will have an independent chair and consist of representatives from the sponsor, patient groups and study partners.

## Data collection, quality and storage

Data will be collected and stored on password protected databases by trial personnel, who are trained in good clinical practice (GCP) and the General Data Protection Regulation (GDPR). Local Principle Investigators will be responsible for ensuring data accuracy and will complete a signed delegation log. Patient reported outcome measures will be completed on paper or using the electronic case report form (ALEA ^TM^) depending on patient preference. All patient reported outcome data will be entered into the electronic case report form (ALEA ^TM^) and data validation will take place according to the procedures set out in the data management plan and data validation plan. Clinical data will be collected from patients’ medical records and entered directly into the electronic case report form (ALEA™) with data validation taking place as per the above statement. Prior to any statistical analysis, all variables will be checked for the number of missing values, impossible values and improbable values. Impossible and improbable values will be defined by clinical opinion. Improbable values will also include values that are outside three standard deviations of the mean value. Any questions regarding the data will go back to the data manager. Descriptive statistics will be calculated for all variables, and distributional assumptions will be checked.

Data collected prior to participant withdrawal or deviation from the protocol will be included, unless participant withdraws consent for its use. Electronic copies of the case report form will be transferred using secure nhs.net email accounts, with data encrypted to ensure anonymity. All procedures for handling, storing, destroying and processing data will be compliant with the Data Protection Act 2018.

All trial documentation and data will be archived centrally by the sponsor at the end of trial in a purpose designed facility for ten years in accordance with regulatory requirements. Access to these archives will be restricted to authorised personnel. Electronic data sets will be stored indefinitely.

## Data monitoring committee

An external, independent Data Monitoring Committee (DMC) will be convened on instruction of the CI and co-investigators on behalf of the project board. It will be made up of experts in the field who are not engaged in any trial activity. The DMC will be responsible for safeguarding the interests of the study participants and assuring the integrity and credibility of the clinical trial.

## Safety reporting

All adverse events are to be recorded in the relevant case report form. Adverse events during CPET are reported to the chief investigator, and adverse events during exercise training (pain and muscle soreness) or psychological support sessions (mental health concerns) are reported to the trial coordinator by the instructor/counsellor and recorded in the relevant case report form by the research physiologist/nurse. Fatal or life-threatening serious adverse events (SAEs) are reported within 24 hours of the local site becoming aware of the event. The SAE form documents the nature of the event, date of onset, severity, corrective therapies given, outcome and causality (that is, unrelated, unlikely, possibly, probably, or definitely). Questions concerning adverse event reporting are directed to the chief investigator in the first instance.

## Conclusion

The Wessex Fit-4-Cancer Surgery trial will be the first pragmatic, robustly conducted randomised controlled study investigating whether a structured and responsive multi-modal exercise training programme +/- psychological intervention will result in a reduced hospital length of stay, improved disability-free survival, reduced in-hospital complications and improved health-related QoL for patients undergoing major inter-cavity resection of cancer.

## Data availability

### Underlying data

No data are associated with this article.

### Extended data

University of Southampton Institutional Repository: The Wessex Fit-4-Cancer Surgery Trial (WesFit): a protocol for a factorial-design, pragmatic randomised-controlled trial investigating the effects of a multi-modal prehabilitation programme in patients undergoing elective major intra–cavity cancer surgery. https://doi.org/10.5258/SOTON/D1790. ^56^

This project contains the following extended data:

- Appendix_3_TIDiER_checklist_for_healthy_conversation_skills_.docx.
- Appendix_2_TIDiER_checklist_for_exercise.docx
- Appendix_4-TIDiER_checklist_for_psychological_support_.docx
- Appendix_5_and_6.docx (Schedule of observations and procedures for primary surgical pathway; and schedule of observations and procedures for neoadjuvant cancer treatment pathway)
- WesFit_readme1.txt

### Reporting guidelines

University of Southampton Institutional Repository: SPIRIT checklist for ‘The Wessex Fit-4-Cancer Surgery Trial (WesFit): a protocol for a factorial-design, pragmatic randomised-controlled trial investigating the effects of a multi-modal prehabilitation programme in patients undergoing elective major intra–cavity cancer surgery’. https://doi.org/10.5258/SOTON/D1790. ^56^

Data are available under the terms of the Creative Commons Attribution 4.0 International license (CC-BY 4.0).

## References

[1] Ho C Kleeff J Friess H : Complications of pancreatic surgery. *HPB.* 2005;7:99–108. 10.1080/13651820510028936 18333171 PMC2023932

[2] Goense L Meziani J Ruurda JP : Impact of postoperative complications on outcomes after oesophagectomy for cancer. *BJS.* 2019;106:111–9. 10.1002/bjs.11000 30370938

[3] McDermott FD Heeney A Kelly ME : Systematic review of preoperative, intraoperative and postoperative risk factors for colorectal anastomotic leaks. *Br J Surg.* 2015 Apr;102(5):462–79. 10.1002/bjs.9697 25703524

[4] Stabenau HF Becher RD Gahbauer EA : Functional Trajectories Before and After Major Surgery in Older Adults. *Ann Surg.* 2019;268(6):911–7. 10.1097/SLA.0000000000002659 29356710 PMC6521949

[5] Lawrence VA Hazuda HP Cornell JE : Functional independence after major abdominal surgery in the elderly. *J Am Coll Surg.* 2004 Nov;199(5):762–72. 10.1016/j.jamcollsurg.2004.05.280 15501119

[6] Moran J Wilson F Guinan E : Role of cardiopulmonary exercise testing as a risk-assessment method in patients undergoing intra-abdominal surgery: A systematic review. *Br J Anaesth.* 2016;116(2):177–91. 10.1093/bja/aev454 26787788

[7] Jack S West MA Raw D : The effect of neoadjuvant chemotherapy on physical fitness and survival in patients undergoing oesophagogastric cancer surgery. *Eur J Surg Oncol.* 2014 Oct;40(10):1313–20. 10.1016/j.ejso.2014.03.010 24731268

[8] West MA Parry MG Lythgoe D : Cardiopulmonary exercise testing for the prediction of morbidity risk after rectal cancer surgery. *Br J Surg.* 2014;101(9):1166–72. 10.1016/j.ejso.2014.03.010 24916313

[9] West MA Loughney L Barben CP : The effects of neoadjuvant chemoradiotherapy on physical fitness and morbidity in rectal cancer surgery patients. *Eur J Surg Oncol.* 2014 Nov;1(11): in press. 10.1016/j.ejso.2014.03.021 24784775

[10] West MA Lythgoe D Barben CP : Cardiopulmonary exercise variables are associated with postoperative morbidity after major colonic surgery: A prospective blinded observational study. *Br J Anaesth.* 2014;112(4):665–71. 10.1093/bja/aet408 24322573

[11] West M Asher R Browning M : Validation of preoperative cardiopulmonary exercise testing-derived variables to predict in-hospital morbidity after major colorectal surgery. *Br J Surg.* 2016;103:744–52. 10.1002/bjs.10112 26914526

[12] West MA Loughney L Lythgoe D : The effect of neoadjuvant chemoradiotherapy on whole-body physical fitness and skeletal muscle mitochondrial oxidative phosphorylation in vivo in locally advanced rectal cancer patients - An observational pilot study. *PLoS One.* 2014;9(12):1–15. 10.1371/journal.pone.0111526 25478898 PMC4257525

[13] Burke S Wurz A Bradshaw A : Physical activity and quality of life in cancer survivors: A meta-synthesis of qualitative research. *Cancers (Basel).* 2017;9(5):1–29. 10.3390/cancers9050053 28531109 PMC5447963

[14] Burke SM Brunet J Sabiston CM : Patients’ perceptions of quality of life during active treatment for locally advanced rectal cancer: The importance of preoperative exercise. *Support Care Cancer.* 2013;21(12):3345–53. 10.1007/s00520-013-1908-2 23912669

[15] Loughney L West MA Dimitrov BD : Physical activity levels in locally advanced rectal cancer patients following neoadjuvant chemoradiotherapy and an exercise training programme before surgery: a pilot study. *Perioper Med.* 2017;6(3):1–8. 10.1186/s13741-017-0058-3 28228938 PMC5311720

[16] Silver JK Baima J : Cancer prehabilitation: An opportunity to decrease treatment-related morbidity, increase cancer treatment options, and improve physical and psychological health outcomes. *Am J Phys Med Rehabil.* 2013;92(8):715–27. 10.1097/PHM.0b013e31829b4afe 23756434

[17] van Rooijen SJ Engelen MA Scheede-Bergdahl C : Systematic review of exercise training in colorectal cancer patients during treatment. *Scand J Med Sci Sport.* 2018;28(2):360–70. 10.1111/sms.12907 28488799

[18] Gillis C Buhler K Bresee L : Effects of nutritional prehabiliation, with and without exercise on outcomes of patients who undergo colorectal surgery: A systematic review and meta-analysis. *Gastroenterology.* 2018;155:391–410. 10.1053/j.gastro.2018.05.012 29750973

[19] Minnella EM Bousquet-Dion G Awasthi R : Multimodal prehabilitation improves functional capacity before and after colorectal surgery for cancer: a five-year research experience. *Acta Oncol (Madr).* 2017;56(2):295–300. 10.1080/0284186X.2016.1268268 28079430

[20] Barberan-Garcia A Ubré M Roca J : Personalised Prehabilitation in High-risk Patients Undergoing Elective Major Abdominal Surgery: A Randomized Blinded Controlled Trial. *Ann Surg.* 2018;267(1):50–6. 10.1097/SLA.0000000000002293 28489682

[21] Treanor C Kyaw T Donnelly M : An international review and meta-analysis of prehabilitation compared to usual care for cancer patients. *J Cancer Surviv.* 2018;12(1):64–73. 10.1007/s11764-017-0645-9 28900822

[22] Piraux E Caty G Reychler G : Effects of preoperative combined aerobic and resistance exercise training in cancer patients undergoing tumour resection surgery: A systematic review of randomised trials. *Surg Oncol.* 2018 Sep;27(3):584–94. 10.1016/j.suronc.2018.07.007 30217322

[23] Tsimopoulou I Pasquali S Howard R : Psychological Prehabilitation Before Cancer Surgery: A Systematic Review. *Ann Surg Oncol.* 2015 Dec;22(13):4117–23. 10.1245/s10434-015-4550-z 25869228

[24] Heldens AFJM Bongers BC Lenssen AF : The association between performance parameters of physical fitness and postoperative outcomes in patients undergoing colorectal surgery: An evaluation of care data. *Eur J Surg Oncol.* 2017 Nov;43(11):2084–92. 10.1016/j.ejso.2017.08.012 28943177

[25] Loughney L West M Kemp G : Exercise interventions for people undergoing multimodal cancer treatment that includes surgery. *Cochrane Database Syst Rev.* 2018;12. 10.1002/14651858.CD012280.pub2 30536366 PMC6517034

[26] Hughes MJ Hackney RJ Lamb PJ : Prehabilitation Before Major Abdominal Surgery: A Systematic Review and Meta-analysis. *World J Surg.* 2019. 10.1007/s00268-019-04950-y 30788536

[27] Macmillan, National institute for health research, Anaesthetists R college of: Principles and guidance for prehabilitation within the management and support of people with cancer. 2019.

[28] Turner J Marthick M Murnane A : Consensus statement on the role of accredited exercise physiologists in the treatment of cancer: A guide for all health professionals involved in the care of people with cancer. 2017.

[29] Cormie P Atkinson M Bucci L : Clinical Oncology Society of Australia position statement on exercise in cancer care. *Med J Aust.* 2018;1. 10.5694/mja18.00199 https://www.mja.com.au/journal/2018/209/6/clinical-oncology-society-australia-position-statement-exercise-cancer-care 29719196

[30] Exercise: The miracle cure and the role of the doctor in promoting it. *Academy of Medical Royal Colleges.* 2015.

[31] West M Loughney L Lythgoe D : Effect of prehabilitation on objectively measured physical fitness after neoadjuvant treatment in preoperative rectal cancer patients: a blinded interventional pilot study. *Br J Anaesth.* 2015 Oct;114(2):244–51. 10.1093/bja/aeu318 25274049

[32] van Rooijen SJ Engelen MA Scheede-Bergdahl C : Systematic review of exercise training in colorectal cancer patients during treatment. *Scand J Med Sci Sports.* 2017; (April):8–13. 10.1111/sms.12907 28488799

[33] Doherty AFO West M Jack S : Preoperative aerobic exercise training in elective intra-cavity surgery: a systematic review. 2013:1–11. 10.1093/bja/aes514 23393151

[34] Valkenet K Van De Port IGL Dronkers JJ : The effects of preoperative exercise therapy on postoperative outcome: A systematic review. *Clin Rehabil.* 2011;25(2):99–111. 10.1177/0269215510380830 21059667

[35] Janssen TL Steyerberg EW Langenberg JCM : Multimodal prehabilitation to reduce the incidence of delirium and other adverse events in elderly patients undergoing elective major abdominal surgery: An uncontrolled before-and-after study. 2019:1–16. 10.1371/journal.pone.0218152 31194798 PMC6564537

[36] Minnella EM Awasthi R Loiselle SE : Effect of Exercise and Nutrition Prehabilitation on Functional Capacity in Esophagogastric Cancer Surgery: A Randomized Clinical Trial. *JAMA Surg.* 2018;153(12):1081–9. 10.1001/jamasurg.2018.1645 30193337 PMC6583009

[37] Levett DZH Grimmett C : Psychological factors, prehabilitation and surgical outcomes: evidence and future directions. *Anaesthesia.* 2019;74:36–42. 10.1111/anae.14507 30604423

[38] Grocott MPWW Browne JP Van der Meulen J : The Postoperative Morbidity Survey was validated and used to describe morbidity after major surgery. *J Clin Epidemiol.* 2007 Sep;60(9):919–28. 10.1016/j.jclinepi.2006.12.003 17689808

[39] CLavien Dindo Dindo D Demartines N Clavien PA : Classification of surgical complications: A new proposal with evaluation in a cohort of 6336 patients and results of a survey. *Ann Surg.* 2004;240(2):205–13. 10.1097/01.sla.0000133083.54934.ae 15273542 PMC1360123

[40] Slankamenac K Graf R Barkun J : The comprehensive complication index: a novel continuous scale to measure surgical morbidity. *Ann Surg.* 2013 Jul;258(1):1–7. 10.1097/SLA.0b013e318296c732 23728278

[41] Herdman M Gudex C Lloyd A : Development and preliminary testing of the new five-level version of EQ-5D (EQ-5D-5L). *Qual Life Res.* 2011;20(10):1727–36. 10.1007/s11136-011-9903-x 21479777 PMC3220807

[42] Aaronson NK Ahmedzai S Bergman B : The European Organization for Research and Treatment of Cancer QLQ-C30: A Quality-of-Life Instrument for Use in International Clinical Trials in Oncology. *J Natl Cancer Inst.* 1993;85(5):365–76. 10.1093/jnci/85.5.365 8433390

[43] Levett DZH Jack S Swart M : Perioperative cardiopulmonary exercise testing (CPET): consensus clinical guidelines on indications, organization, conduct, and physiological interpretation. *Br J Anaesth.* 2018;120(3):484–500. 10.1016/j.bja.2017.10.020 29452805

[44] Adam LM Jarman M Barker M : Use of healthy conversation skills to promote healthy diets, physical activity and gestational weight gain: Results from a pilot randomised controlled trial. *Patient Educ Couns.* 2020 Jun;103(6):1134–42. 10.1016/j.pec.2020.01.001 32035738

[45] Bandura A : *ISocial foundations of thought and action: A social cognitive theory* . NJ1. England: Englewood Cliffs;1986.

[46] McAuley E Blissmer B : Self-efficacy determinants and consequences of physical activity. *Exerc Sport Sci Rev.* 2000 Apr;28(2):85–8. 10902091

[47] Michie S Richardson M Johnston M : The behavior change technique taxonomy (v1) of 93 hierarchically clustered techniques: building an international consensus for the reporting of behavior change interventions. *Ann Behav Med.* 2013;46(1):81–95. 10.1007/s12160-013-9486-6 23512568

[48] Mourtzakis M Prado CMM Lieffers JR : A practical and precise approach to quantification of body composition in cancer patients using computed tomography images acquired during routine care. *Appl Physiol Nutr Metab.* 2008;33(5):997–1006. 10.1139/H08-075 18923576

[49] Martin L Birdsell L MacDonald N : Cancer cachexia in the age of obesity: Skeletal muscle depletion is a powerful prognostic factor, independent of body mass index. *J Clin Oncol.* 2013;31(12):1539–47. 10.1200/JCO.2012.45.2722 23530101

[50] Eisenhauer EA Therasse P Bogaerts J : New response evaluation criteria in solid tumours: Revised RECIST guideline (version 1.1). *Eur J Cancer.* 2009;45(2):228–47. 10.1016/j.ejca.2008.10.026 19097774

[51] Whitehead SJ Ali S : Health outcomes in economic evaluation: The QALY and utilities. *Br Med Bull.* 2010;96(1):5–21. 10.1093/bmb/ldq033 21037243

[52] Singer S Kuhnt S Gotze H : Hospital anxiety and depression scale cutoff scores for cancer patients in acute care. *Br J Cancer.* 2009 Mar;100(6):908–12. 10.1038/sj.bjc.6604952 19240713 PMC2661775

[53] Lorig KR Sobel DS Ritter PL : Effect of a self-management program on patients with chronic disease. *Eff Clin Pract.* 2001;4(6):256–62. 11769298

[54] Hibbard JH Mahoney ER Stockard J : Development and testing of a short form of the patient activation measure. *Health Serv Res.* 2005 Dec;40(6 I):1918–30. 10.1111/j.1475-6773.2005.00438.x 16336556 PMC1361231

[55] Murray E Treweek S Pope C : Normalisation process theory: A framework for developing, evaluating and implementing complex interventions. *BMC Med.* 2010;8. 10.1186/1741-7015-8-63 20961442 PMC2978112

[56] Grimmett C : The Wessex Fit-4-Cancer Surgery Trial (WesFit): a protocol for a factorial-design, pragmatic randomised-controlled trial investigating the effects of a multi-modal prehabilitation programme in patients undergoing elective major intra–cavity cancer surgery. University of Southampton;[Dataset].2021. 10.5258/SOTON/D1790 PMC9490280 36247802

