# Supplementary material for: The Wessex Fit-4-Cancer Surgery Trial (WesFit): a protocol for a factorial-design, pragmatic randomised-controlled trial investigating the effects of a multi-modal prehabilitation programme in patients undergoing elective major intra–cavity cancer surgery
Source: F1000Res. 2022 Aug 2;10:952. Originally published 2021 Sep 21. [Version 2] doi: 10.12688/f1000research.55324.2 (PMC9490280; doi:10.12688/f1000research.55324.2)
Supplement: Peer review history

# Peer review: Reviewer response for version 2

**DOI:** 10.5256/f1000research.136417.r146235

**Published:** 2022-09-20

**Authors:** Celena Scheede-Bergdahl

**Affiliations:** 1 Department of Kinesiology and Physical Education, McGill University, Montreal, QC, Canada

No further comments. Nicely done.

Is the study design appropriate for the research question?

Yes

Is the rationale for, and objectives of, the study clearly described?

Yes

Are sufficient details of the methods provided to allow replication by others?

Partly

Are the datasets clearly presented in a useable and accessible format?

Yes

Reviewer Expertise:

Prehabilitation, cancer, exercise, inflammation

I confirm that I have read this submission and believe that I have an appropriate level of expertise to confirm that it is of an acceptable scientific standard.

# Peer review: Reviewer response for version 2

**DOI:** 10.5256/f1000research.136417.r146236

**Published:** 2022-08-16

**Authors:** Aron Onerup

**Affiliations:** 1 Institute of Clinical Sciences, Sahlgrenska Academy, University of Gothenburg, Gothenburg, Sweden

I have read the authors’ response and feel that they have addressed my previous comments/concerns. I’m satisfied and do not have any further comments.

Is the study design appropriate for the research question?

Yes

Is the rationale for, and objectives of, the study clearly described?

Yes

Are sufficient details of the methods provided to allow replication by others?

Yes

Are the datasets clearly presented in a useable and accessible format?

Yes

Reviewer Expertise:

Prehabilitation with exercise before colorectal cancer surgery.

I confirm that I have read this submission and believe that I have an appropriate level of expertise to confirm that it is of an acceptable scientific standard.

# Peer review: Reviewer response for version 1

**DOI:** 10.5256/f1000research.58889.r96567

**Published:** 2022-02-21

**Authors:** Celena Scheede-Bergdahl

**Affiliations:** 1 Department of Kinesiology and Physical Education, McGill University, Montreal, QC, Canada

This study protocol is nicely put together, by researchers who are obviously very knowledgeable about the area and have experience conducting studies in this field.

 It is very thorough but the only point where I can see where an expansion would be beneficial is the limitation of "cross contamination". Given that information regarding multimodal prehabilitation is widely accessible and compelling, how would the researchers account for those randomized into the control, exercise only or psychological support only groups from participating in their own versions of "multimodal prehabilitation"? 

 This is always worthwhile discussion when we aim to elucidate the various contributing factors to a multimodal approach.

Is the study design appropriate for the research question?

Yes

Is the rationale for, and objectives of, the study clearly described?

Yes

Are sufficient details of the methods provided to allow replication by others?

Partly

Are the datasets clearly presented in a useable and accessible format?

Yes

Reviewer Expertise:

Prehabilitation, cancer, exercise, inflammation

I confirm that I have read this submission and believe that I have an appropriate level of expertise to confirm that it is of an acceptable scientific standard, however I have significant reservations, as outlined above.

# Response

**Published:** 2022-07-20

**Authors:** Chloe Grimmett

**Affiliations:** University of Southampton, UK

Thank you for the review and apologies for the delay in replying. We were not aware that replies to your comments were still outstanding, please accept our sincere apologies.

 Thank you for your kind comments.

 Control group contamination is a real issue, but luckily not seen in any of our cohorts. The reason being is that the control groups also benefit from activity advice, nutrition advice, and surgery school which are all standard of care. In our smaller cohorts we have given patients activity monitors to objectively measure patients’ activity and statistically account for it if contamination is seen; however, it was felt that this cohort was too large to undertake this in our usual manner. With that said, however, one of our exploratory outcomes will be to look for control group contamination using a sub-sample of participants recruited to all four groups. We will be undertaking this by a five-day objectively measured in-depth analyses of activity (repeatable over three time points) as we have done on our smaller cohorts (all referenced). Moreover, we have explored this in other cohorts and we have found that even if patients improve PA, unfortunately, this doesn’t translate to improvement in fitness or even outcome. This, we think, is down to the nature of the high-interval training programme which cannot be substituted for an improvement in PA.

# Peer review: Reviewer response for version 1

**DOI:** 10.5256/f1000research.58889.r101805

**Published:** 2021-12-22

**Authors:** Aron Onerup

**Affiliations:** 1 Institute of Clinical Sciences, Sahlgrenska Academy, University of Gothenburg, Gothenburg, Sweden

The authors have planned a rational and interesting study. I have some suggestions for the authors to consider in order to optimize the output from their efforts and some minor comments on the text:

1. The authors write in the conclusions in the abstract that this is the first RCT of its kind. I do not find this to be correct, and I do not find it to be necessary. Carli *et al.* ^1^ have evaluated multimodal rehabilitation before colorectal cancer surgery. I would consider the main strength of this study is that it includes high-intensity training, a large sample size, and clinically relevant outcomes.
2. The authors have performed several previous studies on the association between fitness and outcomes after surgery. It would be interesting if the authors could provide a pathophysiological rationale for the study with preliminary observational results on the association between both fitness and physical activity and postoperative outcomes in order to motivate the chosen outcomes, rather than a list of previous studies. This could also include more recent articles, e.g. the SR by Steffens *et al.* ^2^ and the article by Onerup *et al.* ^3^.
3. For colorectal cancer surgery, I would say that there are three well-performed RCTs where the effect of exercise has been evaluated for clinically relevant outcomes: Barberan-Garcia *et al.* ^4^, Carli *et al.* ^1^, and our pragmatic RCT recently reported, Onerup *et al.* ^5^. I suggest the authors add these articles to the background since it has some common elements with the current study, albeit with a higher intensity (interesting) intervention in the current study.
4. Given the results from Barberan-Garcia *et al.* ^4^, Onerup *et al.* ^5^ *,* and Onerup *et al.* ^3^, it is reasonable to believe that the exercise intervention will have the most effect on non-surgical morbidity, e.g. cardiovascular complications, pulmonary complications, and possibly infections, etc. Have the authors considered focusing on, or at least measuring, these outcomes rather than general outcomes (length of hospital stay and CCI) mostly driven by surgical complications, where it is less probable that the intervention will have an effect? I fear that the authors risk missing a large portion of the effect with their chosen outcome measures.
5. I appreciate the pragmatic design. Have the authors considered rating the pragmatic design using the tool described in BMJ?
6. Regarding randomisation, I understand it as 4 treatment groups, stratified for: tumor type (8 alternatives), hospital site (unknown number), gender (2 alternatives), neoadjuvant therapy (≥2 alternatives), and age (≥2 alternatives). This gives me at least 4x8x2x2x2=256 x the number of hospitals strata. Suddenly the planned sample size sounds relatively small. When adding the wide spectrum of postoperative recovery for the various tumor types, I wonder if the authors consider the sample size to be large enough to find clinically relevant differences. Will the population be analyzed as one cohort or as different cohorts depending on tumor type?

In conclusion, the authors should be commended for performing the described study and I hope that my suggestions/questions may improve the outcome of the study.

Is the study design appropriate for the research question?

Yes

Is the rationale for, and objectives of, the study clearly described?

Yes

Are sufficient details of the methods provided to allow replication by others?

Yes

Are the datasets clearly presented in a useable and accessible format?

Yes

Reviewer Expertise:

Prehabilitation with exercise before colorectal cancer surgery.

I confirm that I have read this submission and believe that I have an appropriate level of expertise to confirm that it is of an acceptable scientific standard, however I have significant reservations, as outlined above.

## References

[1] : Effect of Multimodal Prehabilitation vs Postoperative Rehabilitation on 30-Day Postoperative Complications for Frail Patients Undergoing Resection of Colorectal Cancer. *JAMA Surgery* .2020;155(3) : 10.1001/jamasurg.2019.5474 10.1001/jamasurg.2019.5474 PMC699065331968063

[2] : Is preoperative physical activity level of patients undergoing cancer surgery associated with postoperative outcomes? A systematic review and meta-analysis. *Eur J Surg Oncol* .45(4) : 10.1016/j.ejso.2018.10.063 510-518 10.1016/j.ejso.2018.10.063 30910052

[3] : Association between self-assessed preoperative level of physical activity and postoperative complications - An observational cohort analysis within a randomized controlled trial (PHYSSURG-C). *Eur J Surg Oncol* .2021; 10.1016/j.ejso.2021.10.033 10.1016/j.ejso.2021.10.033 34742613

[4] : Personalised Prehabilitation in High-risk Patients Undergoing Elective Major Abdominal Surgery: A Randomized Blinded Controlled Trial. *Ann Surg* .2018;267(1) : 10.1097/SLA.0000000000002293 50-56 10.1097/SLA.0000000000002293 28489682

[5] : Effect of Short-Term Homebased Pre- and Postoperative Exercise on Recovery after Colorectal Cancer Surgery (PHYSSURG-C): A Randomized Clinical Trial. *Ann Surg* .2021; 10.1097/SLA.0000000000004901 10.1097/SLA.0000000000004901 33843798PMC8820776

# Response

**Published:** 2022-07-19

**Authors:** Chloe Grimmett

**Affiliations:** University of Southampton, UK

Thank you for the review and apologies for the delay in replying. We were not aware that replies to your comments were still outstanding, please accept our sincere apologies.

 1) We have removed the 'first' in the abstract and concentrated on the high-intensity rehabilitation as the novelty in this study.

 2) We have included the suggested references, thank you, and have elaborated more on the data in the introduction.

 3) We have included a section on recent RCTs recommended showing illustrating mixed outcomes in high-quality trials.

 4) Indeed, the reviewer is right. The primary outcome measure is a commissioner-driven metric along with all the patient-reported outcome metrics (WHO-DAS, EQ-5D, etc.) and will give us an in-depth understanding of heath economical endpoints which are imperative for service delivery in this area. Complications will be measured in 3 ways (Clavien-Dindo, POMS 18-item, and CCI). POMS-18 items measured on days 3, 5, 7, and 15 will ensure capture of complications in nine distinct domains including but not restricted to complications shown to be improved by prehab in the various RCTs we included in the introduction.

 5) No, unfortunately, we were unaware that such a tool existed. WesFit is currently actively recruiting, so we are unable to change our trial methodology at this stage.

 6) Indeed, the strata are significant, however, such a large pragmatic trial also needs to be deliverable. Increasing the number of patients to account for each stratum would make this trial unfeasible. We feel that the sample size calculation we undertook is adequate. As described, the sample size was based on the two main comparisons (i.e. exercise vs. no exercise and psychological support vs. no support). The trial sample size is the larger of these two comparisons, so in this case, the sample size calculation is powered on the psychological support comparison. The sample size calculation assumes that there is no interaction between the interventions. The population will be analysed as one cohort.
